# Protein kinase R-like endoplasmatic reticulum kinase is a mediator of stretch in ventilator-induced lung injury

**DOI:** 10.1186/s12931-018-0856-2

**Published:** 2018-08-22

**Authors:** Tamás Dolinay, Chanat Aonbangkhen, William Zacharias, Edward Cantu, Jennifer Pogoriler, Alec Stablow, Gladys G. Lawrence, Yoshikazu Suzuki, David M. Chenoweth, Edward Morrisey, Jason D. Christie, Michael F. Beers, Susan S. Margulies

**Affiliations:** 1 Department of Medicine, Division of Pulmonary, Allergy and Critical Care Medicine, University of Pennsylvania, 3400 Spruce St, Philadelphia, PA 19104 USA; 2 Department of Medicine, Division of Pulmonary and Critical Care Medicine, University of California Los Angeles, 10833 Le Conte Ave, Los Angeles, CA 90095 USA; 3 Department of Chemistry University of Pennsylvania, 231 S 34th St, Philadelphia, PA 19104 USA; 4 Department of Surgery, University of Pennsylvania, 3400 Spruce St, Philadelphia, PA 19104 USA; 5 Department of Pathology, Children’s Hospital of Philadelphia, 3400 S 34th St, Philadelphia, PA 19104 USA; 6 Department of Bioengineering, University of Pennsylvania, 210 South 33rd St, Suite 240 Skirkanich Hall Philadelphia, Philadelphia, PA 19104 USA; 7 Department of Medicine, Division of Cardiovascular Medicine, University of Pennsylvania, 3400 Spruce St, Philadelphia, PA 19104 USA; 8 Wallace H. Coulter Department of Biomedical Engineering Georgia Institute of Technology and Emory University, University School of Medicine, U.A. Whitaker Building, 313 Ferst Drive, Suite 2116, Atlanta, GA 30332-0535 USA

**Keywords:** Ventilator-induced lung injury, Protein kinase R-like endoplasmic reticulum kinase, Alveolar epithelium

## Abstract

**Background:**

Acute respiratory distress syndrome (ARDS) is a severe form of lung injury characterized by damage to the epithelial barrier with subsequent pulmonary edema and hypoxic respiratory failure. ARDS is a significant medical problem in intensive care units with associated high care costs. There are many potential causes of ARDS; however, alveolar injury associated with mechanical ventilation, termed ventilator-induced lung injury (VILI), remains a well-recognized contributor. It is thus critical to understand the mechanism of VILI. Based on our published preliminary data, we hypothesized that the endoplasmic reticulum (ER) stress response molecule Protein Kinase R-like Endoplasmic Reticulum Kinase (PERK) plays a role in transmitting mechanosensory signals the alveolar epithelium.

**Methods:**

ER stress signal responses to mechanical stretch were studied in ex-vivo ventilated pig lungs. To explore the effect of PERK inhibition on VILI, we ventilated live rats and compared lung injury parameters to non-ventilated controls. The effect of stretch-induced epithelial ER Ca^2+^ signaling on PERK was studied in stretched alveolar epithelial monolayers. To confirm the activation of PERK in human disease, ER stress signaling was compared between ARDS and non-ARDS lungs.

**Results:**

Our studies revealed increased PERK-specific ER stress signaling in response to overstretch. PERK inhibition resulted in dose-dependent improvement of alveolar inflammation and permeability. Our data indicate that stretch-induced epithelial ER Ca^2+^ release is an activator of PERK. Experiments with human lung tissue confirmed PERK activation by ARDS.

**Conclusion:**

Our study provides evidences that PERK is a mediator stretch signals in the alveolar epithelium.

## Background

Alveolar epithelial injury is a well-recognized complication of injurious mechanical ventilation [1, 2]. Experimental data show that repetitive overdistention of the alveolar wall by high tidal volumes, also referred to as overstretch, results in damage to the epithelial barrier with subsequent inflammation and pulmonary edema formation. This injury, termed ventilator-induced lung injury (VILI), results in pathological changes similar to what is seen in acute respiratory distress syndrome (ARDS) [3–5]. While it is difficult to discern VILI in ventilated patients [1, 6], clinical studies show that overstretch of the alveolar wall contributes to the development of ARDS [7, 8]. As ARDS remains a significant medical problem in intensive care units [2], understanding the effect of stretch on epithelial barrier function is critical. Our laboratory and others have identified mechanosensory mechanisms in the alveolar epithelium that regulate epithelial barrier function [9–12]. Most recently, we have shown that the Protein Kinase R-like Endoplasmic Reticulum Kinase (PERK)-mediated Integrated Stress Response (ISR) intracellular signaling pathway is important in transmitting these mechanical stress signals in the alveolar epithelium [13], but the role of PERK in VILI remains unknown.

PERK along with Inositol Requiring Enzyme (IRE)-1α and Activating Transcription Factor (ATF)-6, is a sensor of endoplasmic reticulum (ER) stress signals [14]. While the three sensory mechanisms are interconnected [15], PERK specifically responds to mechanical stretch in the alveolar epithelium [13]. At baseline condition, PERK is bound to Binding immunoglobulin Protein (BiP) in the ER membrane in its inactive dephosphorylated form. Upon stress signal PERK autophosphorylates and via its kinase domain phosphorylates eukaryotic Initiation Factor-2α (eIF2α), which is the rate limiting step of ISR activation. This causes the temporary cessation of eIF2 complex-mediated protein translation [15]. In addition to a general decrease in protein translation, transcription factors such as Activating Transcription Factor 4 (ATF4) and CCAAT/Enhancer-binding Protein Homologous Protein (CHOP) are selectively translated to promote RNA transcription. Temporary activation of ISR via ATF4 results in the activation of “pro-cell survival” gene transcription e.g., Growth Arrest and DNA Damage-inducible Protein (GADD)-34; which then restores protein synthesis by the dephosphorylation of eIF2α [16]. Sustained activation of ISR leads to CHOP activation with gene transcription resulting in cellular dysfunction, death and activation of the immune system [17, 18].

Previously, we reported causal links between ISR activation and mechanical stretch, revealing a novel mechanism of VILI signaling. In this study we extended our initial observations to a clinically relevant VILI model and human ARDS and show that PERK, the upstream regulator of ISR, is a critical mediator of mechanical stretch in the alveolar epithelium. The inhibition of PERK signaling offers a new pathway to mitigate VILI and to prevent ARDS.

## Methods

### Human lung sample collection and analysis

Lung tissue samples (*N* = 9) were received from the Prospective Registry of Outcomes in Patients Electing Lung Transplantation (PROPEL) bio specimen repository and clinical database (Biobank). This study approved by the IRB of the University of Pennsylvania under protocol number 813685. The Biobank regularly receives lung tissue from donors and recipients of lung transplants. Many donated lungs are declined for transplantation for clinical reasons and these tissues become available for research. We amended protocol 13643/00, Cellular injury mediators of acute respiratory distress syndrome in human lung tissue, to PROPEL, to receive lung tissue samples and limited clinical information on lung tissue samples used for our analysis. Lung tissue samples were collected by the investigators of the current study immediately after the lungs have arrived to the University of Pennsylvania. Lungs were stored on +4C during transportation and they have become available to us within 24 h after harvest. After visual inspection, lung tissue samples were resected from right upper and middle lobes. Samples were used for total protein extraction. Available lung histology report and limited clinical information including age, gender, date of collection, date of death, last available arterial partial oxygen pressure (paO2 in mmHg) within 24 h, inspired percent of oxygen via mechanical ventilation and admission diagnosis was obtained for all samples (Table 1).

**Table 1:** Human lung tissue sample clinical, radiographic and pathological characteristics

ID	Age	Gender	Race	B/l infiltrate	P/F	Ventilation (days)	Alveolar pathology			Admit Dx	ARDS
							neutrophils	septal edema	hyaline membrane (DAD)		
1	27	M	W	N	105	2	N	N	N	PE	N
2	68	M	W	N	204	3	N	N	N	COPD	N
3	34	M	AA	N	35	4	N	N	N	COPD	N
4	58	F	AA	N	160	3	N	Y	N	pulm. fibrosis	N
5	25	M	W	Y	55	2	Y	Y	N	PNA	Y
6	47	M	W	Y	182	2	Y	N	N	COPD	Y
7	68	F	W	Y	160	4	Y	N	Y	PNA	Y
8	28	M	W	Y	65	6	Y	N	Y	PNA	Y
9	53	M	W	Y	75	16	Y	N	Y	COPD	Y

*B/l* Acute bilateral infiltrate on imaging, *P/F* a ratio of arterial oxygen partial pressure in mmHg to fractional inspired oxygen (FiO_2),_ *DAD* diffuse alveolar damage, *Dx* principal diagnosis, *PE* pulmonary embolism, *COPD* chronic obstructive lung disease, *pulm* pulmonary, *PNA* pneumonia, *ARDS* acute respiratory distress syndrome, *Y* condition present, *N* condition not present

### ARDS definition in human samples

We used a combination of available clinical, radiological and histological parameters to diagnose ARDS. Only samples obtained from endotracheally intubated patients were eligible to allow precise measurement of fractional inspired oxygen (FiO_2_). The presence of acute bilateral lung infiltrate on imaging within 1 week prior to death, a ratio of arterial oxygen partial pressure in mmHg to FiO_2_ less than 300 and a pathological evidence neutrophilic infiltrates or hyaline membranes in the alveolar space with or without septal edema. Histological analysis of samples was performed on H&E stained slides by a pathologist blinded to the clinical history of the patient. The presence of diffuse alveolar damage (DAD) on pathology was not a strict criterion for ARDS as its absence does not definitely rule out the disease [19, 20]. Samples not meeting this criteria were used as non-ARDS controls (Table 1).

### Ex vivo pig lung perfusion, ventilation experiment

Pig heart-lung complexes (*N* = 12) were obtained from euthanized animals used as untreated controls in Preclinical Therapy Trials for Pediatric TBI study. All animals are housed in accordance with guidelines from the American Association for Laboratory Animal Care and Research. The protocol was approved by the Institutional Animal Care Use Committee at the University of Pennsylvania under protocol number 803401. Heart-lung complexes including the trachea were harvested *en block* from Yorkshire piglets (age 4 weeks, weight 6–10 kgs, *N* = 12) post mortem. The pulmonary circulation was flushed via the right ventricle using 1 l sterile normal saline. The heart was carefully removed and the pulmonary trunk was preserved. A 16 G angiocatheter was sutured in the pulmonary artery and connected to a peristaltic surgical pump via tubing. A 3 mm diameter endotracheal tube was inserted in the trachea and sutured in place. The complex was placed in a 37 C heated and humidified chamber hanging by the endotracheal tube. The lungs were perfused with 0.3 L/min MEM supplemented with 3% FBS as explained for the human perfusion-ventilation model. The lung complexes were subsequently randomized in three groups: no stretch (NS), low-stretch (LS) 6 ml/kg tidal volume (Vt) positive pressure ventilation and overstretch (OS) ventilation (Vt = 12 ml/kg) for 4 h using volume control ventilation (Puritan Bennett 7200 ventilator) with 5 cmH2O applied positive end expiratory pressure (PEEP) and FiO2 of 50%. We used 50% FiO2 to match the experimental conditions used in our in vivo rat model. At the end of the experiment the right lower lobe was perfused with 10% formalin and used for histology and the left lower lobe was used for total protein extraction and western blotting as described in lung tissue protein studies.

### Pig and human lung tissue protein analysis

Tissue pieces (1 cm by 1 cm) were submerged in at least 5-fold volume of cold RIPA buffer and homogenized with Dounce homogenizer. Samples were centrifuged for 20 min at 13,000 RPM on tabletop centrifuge in 1.0 ml aliquots (Beckman Microfuge R, Backman Instruments, Palo Alto, CA). The supernatant was used to determine total protein concentration using colorimetric BioRad Protein DC assay (BioRad, Hercules, CA). Sample protein contents were compared to standard protein curves using KC Junior spectrophotometer plate reader (BioTek Instruments Winooski, VT) at 750 nm wavelength. Equal protein was loaded on precast 4–12% Bis-Tris gels (Invitrogen NuPage Novex mini gels, Life Technologies, Carlsbad, CA). Standard western blotting was performed for Binding immunoglobulin Protein (BiP), phosphorylated (p) and total (t) forms of Protein Kinase R-like Endoplasmic Reticulum Kinase (PERK) and eukaryotic Initiation Factor (eIF)-2α, Activating Transcription Factor (ATF)-4 and 6, CCAAT/Enhancer-binding Protein Homologous Protein (CHOP), Growth Arrest and DNA Damage-inducible Protein (GADD)-34 and X-box Binding Protein (XBP)-1. We used total (t)-forms of PERK and eIF2α to normalize band densities of phospho (p)-forms. For all other proteins β-actin was used as loading control (Santa Cruz Biotechnologies, Santa Cruz, CA). The following primary antibodies were used: BiP (A-10) mouse monoclonal antibody (Santa Cruz), p-PERK (Abcam, Cambridge, MA) and t-PERK (Cell Signaling Technology, Danvers, MA), p-eIF2α (Ser 51) and t-eIF2α rabbit monoclonal antibody (Cell Signaling), ATF4 rabbit polyclonal antibody (Sigma-Aldrich Company, Saint Louis, MO), ATF6 (N-terminal) rabbit polyclonal antibody (Aviva Systems Biology, San Diego, CA), CHOP (F-168) mouse polyclonal antibody (Santa Cruz), GADD34 rabbit polyclonal antibody (Proteintech Group Inc., Rosemont, IL), XBP-1 rabbit monoclonal antibody (Abcam, Cambridge, MA) and B-actin mouse polyclonal antibody (Santa Cruz). In pig tissue studies, the protein levels of *N* = 4 samples from no stretch, low-stretch and overstretch conditions were compared with western blotting. In human tissue studies, the protein levels of N = 4 of controls and *N* = 5 of ARDS samples were compared with western blotting. Blots were rinsed with 0.1% Tween 20 containing tris buffered saline (TBST) and incubated with secondary antibodies against rabbit or mouse diluted in 5% milk solution. Blots were rinsed again with TBST treated with high-sensitivity western blot substrate (Pierce ECL Plus, ThermoFisher Scientific, Waltham, MA). High sensitivity autoradiographic film was used to detect chemiluminescence. Immunoblots were scanned and bend densities were analyzed using FIJI software [21].

### Pig tissue immunofluorescence studies

For immunofluorescence (IF) analysis antibodies p-PERK (#192591, Abcam) and occludin (Invitrogen # 33–1500, ThermoFisher Scientific) were used on formalin-fixed, paraffin–embedded pig lung tissue. Paraffin was cleared with xylene, and slides were rehydrated through descending concentrations of ethanol. Slides were then treated with 3% H2O2/methanol for 30 min. Slides were pretreated in a pressure cooker (Biocare Medical, LLC, and Concord, CA) in Antigen Unmasking solution (H3300, Vector Laboratories, Burlingame, CA). After cooling, slides were blocked in Sudan Black (199664-25G, Sigma-Aldrich) for 20 min at RT. Slide were then rinsed in 0.1 M Tris Buffer, then blocked with 2% fetal bovine serum for 15 min. Slides were then incubated with p-PERK at 1:200 dilution for 1 h at room temperature. Slides were again rinsed, then incubated with anti-rabbit polymer secondary prediluted (K4003, DAKO, Carpenteria, CA) for 30 min at room temp. After rinsing, slides were then incubated with the TSA biotin complex (NEL7490B001, PerkinElmer, Waltham, MA) at 1:50 for 10 mins at room temp. Slides were then rinsed and incubated with Alexa 488 Streptavidin (green) secondary (A21370, Life Technologies, Eugene, OR) 1:200 for 30 min at room temp. After rinsing, slides were then treated in preheated 5% SDS (CS-5585-28, Denville Scientific, Metuchen, NJ) for 7 min at 55C. Slides were then rinsed and blocked with 2% fetal bovine serum again before incubating with occludin at 1:100 for 1 h at room temp. After rinsing slides were incubated for 1 h in Alexa 594 (red) anti-rabbit secondary (A11012, Life Technologies, Eugene, OR). Slides were then rinsed, counterstained with DAPI, and rinsed again before cover slipping with Prolong Gold (P36930, Life Technologies, Eugene, OR). Fluorescence was detected with Zeiss LSM 880 laser scanning confocal microscope using Plan Apo 20X and 40X objective with NA = 0.8. Pictures were taken of 5 independent areas. Alexa 488 positive cells (p-PERK) were count in 100 Alexa 594 positive cells (occludin) per field and the number of dual positive cells was compared between non-ventilated and ventilated lung tissue. Images were analyzed using Fiji imaging software [21] .

### Rat experiments

Adult male Sprague-Dawley rats (*N* = 94, weight 250–300 g) were purchased from Charles River Laboratories (Horsham, PA). Rats were allowed to acclimate for 1 week with rodent chow and water ad libitum. All animals are housed in accordance with guidelines from the American Association for Laboratory Animal Care and Research. Protocols were approved by the Institutional Animal Care Use Committee at the University of Pennsylvania (Protocol numbers 805201 and 805630).

### A. Freshly isolated alveolar epithelial cell experiments

#### Alveolar epithelial cell isolation, transfection and AEC-I monolayer preparation

The protocol for cell isolation and culture has been previously described by us in detail [13]. In brief, the lungs were removed *en bloc* from euthanized rats (*N* = 10)**.** The lung tissue was digested with elastase (Worthington Biochemical Corporation, Lakeside, NJ) and filtered to obtain a single cell suspension. Next, epithelial cells were isolated using negative selection using rat IgG coated plates containing serum-free media. The remaining cells were collected. Our isolation method yields approximately 95% pure alveolar epithelial type–II (RAEC-II) cells. Cells were plated on fibronectin-coated custom made silicone membranes. We plated 1.3 million cells on a 1 cm diameter membrane. We usually collect 30–50 million AEC-II cells per rat, which allows us to prepare 32–40 monolayers per animal. Cells were cultured with 10% FBS-containing Minimal Essential Medium (MEM) containing antibiotics for 4 days to obtain monolayers. Rat AEC-II cells transform to alveolar epithelial type-1 like (RAEC-I) cells during this period of time and express only AEC-I cell surface markers. Over the course of 4 days they form a monolayer impermeable to large molecules [22]. On the day of the experiment, the medium was removed and replaced with HEPES-buffered MEM.

#### Cyclic cell stretch

Cell stretch was performed in a 37C incubator. RAEC-I monolayers were stretched (S) or used as unstretched controls (NS) in groups of 8 to obtain sufficient material for further analysis. *N* = 4 biological replicates per conditions were used. Cells underwent biaxial 15/min cyclic stretch with 25% surface change on our custom-built cell stretcher for 1, 3 and 6 h. Groups of monolayers were pretreated with 20 μM Ryanodine (Torcis Biosciences, Minneapolis, MN, Ry) to study the effect of Ca^2+^ signaling inhibition on stretch. Monolayers were used for immunofluorescence analysis.

#### Epithelial monolayer immunofluorescence studies

At the end of the experiment, RAEC-I monolayers were fixed on the flexible membranes with 4% formaldehyde, permeabilized with 0.5% Triton X and incubated with p-PERK primary antibody (#192591, Abcam, 1:200 dilution) for 45 min. Following washing with 0.1% Tween containing PBS the monolayers were incubated Alexa 488 Streptavidin (green) secondary (A21370, Life Technologies) antibody for 20 mins and counterstained with DAPI. Membranes were transferred on glass slides to facilitate analysis with confocal microscopy. Green fluorescence was compared among conditions using confocal microscopy as described in pig IF studies. Pictures were taken of 5 independent areas and green fluorescence was compared between control and stretched monolayers. Fluorescent intensity was quantified using Fiji imaging software [21].

#### Epithelial monolayer protein analysis

Total protein was extracted from groups of monolayers for each condition using RIPA buffer. Western immunoblotting was performed for p- and t-eIF2α as explained in lung tissue analysis. Band densities for *N* = 4 biological replicates per condition were analyzed with FIJI.

### B. In vivo rat VILI experiments

#### In vivo PERK inhibitor treatment and mechanical ventilation protocol

Rats (*N* = 84) received 3, 10 or 30 mg/kg PERK inhibitor GSK 2606414 (PI) or vehicle control 0.1% Tween 80 + 0.5% hydroxypropyl-methylcellulose (VC) via oral gavage 4 h before the start of mechanical ventilation in 2.5 ml volume bolus. Subsequently, animals were randomized to receive mechanical ventilation, or they were let to breathe spontaneously. To control for the off target effects of PI, groups of spontaneously breathing animals received 30 mg/kg PI. *N* = 9/group were used for BAL studies and 5/group for endo/epithelial permeability studies. For the mechanical ventilation protocol, rats were kept under general anesthesia for the entire procedure. Their breathing, cardiac function and neurological responses were continuously monitored to minimize pain and distress. Induction and maintenance anesthesia was performed with inhaled 2–4% isoflurane. If additional anesthesia was necessary to keep the animal in surgical plane at the time of induction, it was given an intraperitoneal injection of a mixture of ketamine (75–100 mg/kg) and xylazine (5-10 mg/kg). To minimize injectable anesthesia and improve analgesia use, we injected the subcutaneous tissue of the tracheostomy site with 1% lidocaine. To ensure an adequate level of anesthesia, animals were continuously monitored for heart rate and blood pressure with the CODA non-invasive blood pressure tail cuff (Kent Scientific, Torrington, CT), and consciousness was monitored with a toe pinch every 15 min. We used heat blanket to maintain ambient temperature of 38C throughout the experiment. Our VILI protocol was previously described [13]. In brief, the rats were tracheotomized; a 14-gauge metal cannula was placed in their trachea and sutured tight. Animals were mechanically ventilated for 4 h with 20 ml/kg tidal volume with an Inspira advanced safety rodent ventilator with 50% FiO2 (Harvard Apparatus, Holliston, MA). No positive end expiratory pressure (PEEP) was added, and no recruitment maneuvers were performed to maximize injury. At the end of the experiment, euthanasia was performed by thoracotomy and transection of great vessels.

#### E. Bronchoalveolar lavage and sample analysis

We obtained bronchoalveolar lavage (BAL) following euthanasia, the chest cavity was opened with a midline incision from all animals groups (*N* = 54, 9 animals/group). The right main stem bronchus was isolated, and a suture was placed around the bronchus. The left lung was lavaged with 3 ml cold PBS. BAL samples were centrifuged at 5000 RPM for 10 min to separate cells from supernatant. The cell pellet was used for quantitative and qualitative cell counts. Cells were reconstituted in 1 ml PBS. Total BALF cell counts were obtained using a hemocytometer (Hausser Scientific, Horsham, PA). For differential cell count, 100 μl BALF was mounted on a glass slide by centrifuging the fluid at 1000 RPM for 10 min (Cytospin 2, Shandon Scientific, Runcorn, Cheshire, United Kingdom). Slides were stained with hematoxylin and eosin (H&E) and 200 cells were counted.

#### F. Endo/epithelial permeability measurement with FITC-labeled albumin extravasation

This protocol was previously described by us [13]. In brief, a separate group of animals (*N* = 30, 5 animals/group) were injected with 1 mg/animal fluorescent isothiocyanate (FITC)-labeled bovine albumin (Sigma-Aldrich) diluted in 0.1 ml sterile saline 1 h before sacrifice via the tail vein. Following sacrifice, undiluted BAL and plasma were collected. The fluorescence of 100 μl of BAL and 1:10 diluted plasma was measured at 488 nm excitation and 520 nm emission wavelengths in duplicates (Cytofluor 4000 fluorescent plate reader, Perseptive Biosciences, Framingham, MA). The ratio of BAL to plasma fluorescence was used to express permeability [13].

#### Statistics

For comparative studies of densitometry, cell count and permeability studies we used the Kruskal-Wallis test for multi-group comparisons and intergroup differences were analyzed with the Wilcoxon rank sum test. For fluorescent image intensity analysis, we used two-way ANOVA with Tukey-Kramer post hoc analysis (JMP software, Cary, NC). Results are presented as mean ± SEM. Significance level was *P* < 0.05.

## Results

### Mechanical overstretch selectively activates PERK ER stress signaling in porcine lung tissue

**Fig. 1:**
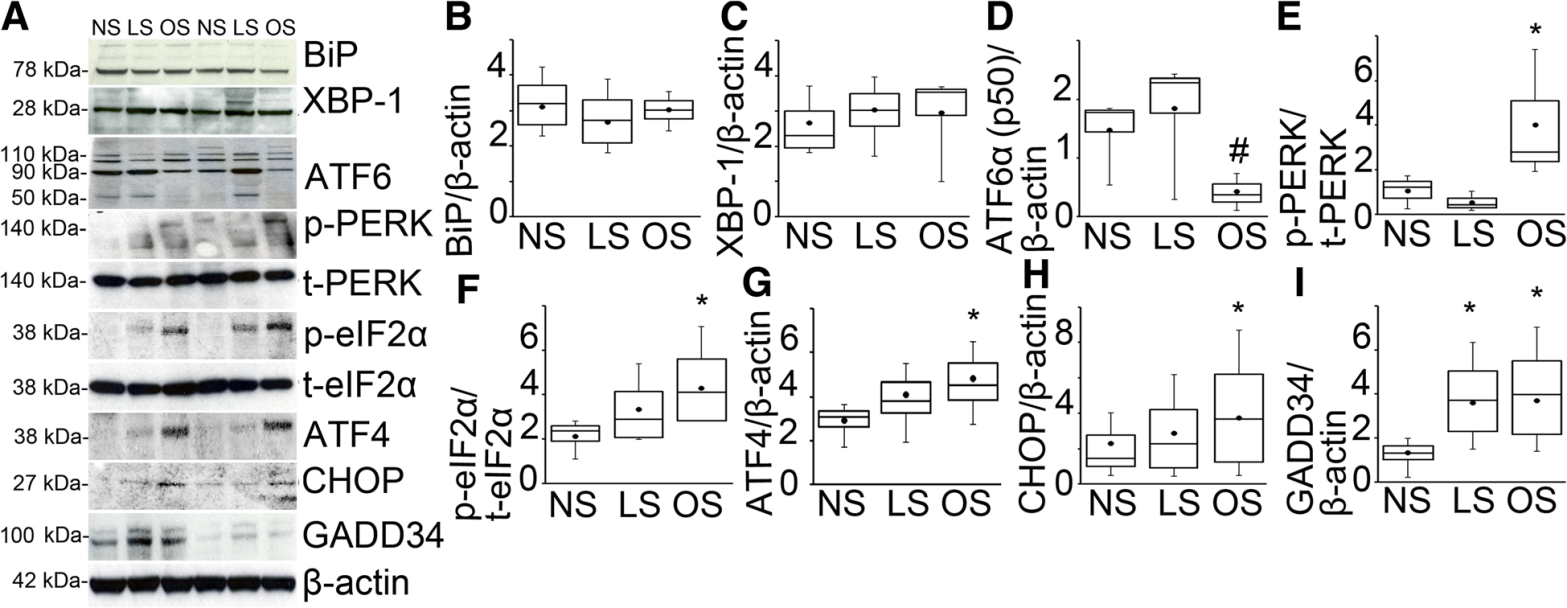
Mechanical overstretch selectively activates PERK ER stress signaling in porcine lung tissue. **a**. Perfused porcine lung complexes were prepared and randomized to receive low (LS, Vt = 6 ml/kg) or high stretch (overstretch, OS, Vt = 12 ml/kg) mechanical ventilation for 4 h or used as non-ventilated controls (NS). ER stress signal pathways were detected in extracted total protein using western blotting. Stretch did not affect Binding Immunoglobulin Protein (BiP) and X-box Binding Protein (XBP)-1 protein expression. OS significantly decreased the expression of cleaved form of Activating Transcription Factor (ATF) 6α (p50) compared to NS. We detected the activation of Protein Kinase RNA-like Endoplasmic Reticulum Kinase (PERK), eukaryotic Initiation Factor (eIF)-2α and the increased expression of downstream Integrated Stress Response proteins ATF4, CCAAT/Enhancer-binding Protein Homologous Protein (CHOP) and Growth Arrest and DNA Damage-inducible Protein (GADD)-34 expression. We used t-PERK for loading control of p-PERK, t-eIF2α for p-eIF2α and β-actin for other proteins. **b**-**i**. Quantified data is shown. Statistics: Kruskal-Wallis test was performed for multiple group comparison, and intergroup differences were analyzed with Wilcoxon rank sum test. *N* = 4 animals/group, *p* < 0.05. Data is presented as averaged values ±SEM in a five number summary format. * represents significant increase in p-PERK, p-eIF2α, ATF4, CHOP and GADD34 in OS and LS vs C conditions. # represents significant decrease in ATF6α expression in OS vs. NS conditions. The same abbreviations will be used in subsequent figures

**Fig. 2:**
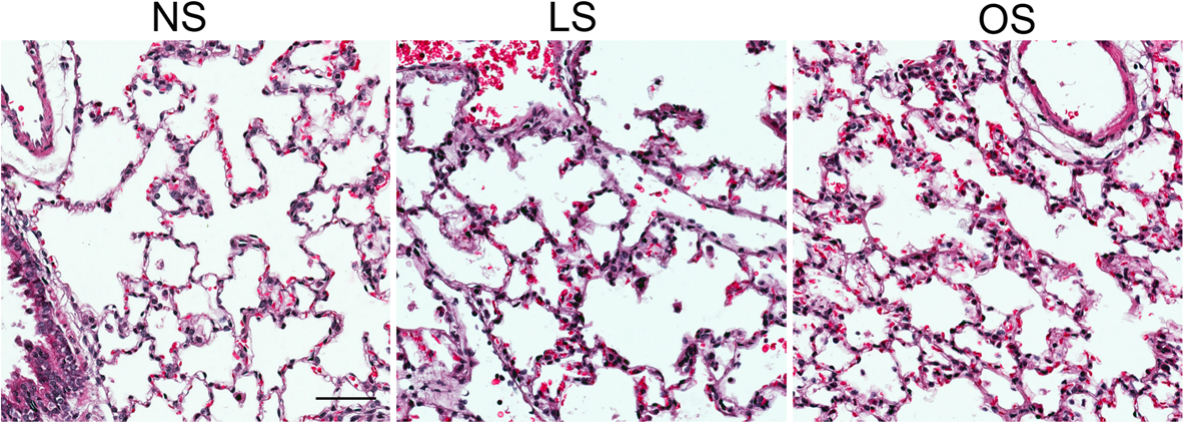
Histology analysis of ventilated pig lung tissue. Mechanical ventilation with low stretch (LS, 6 ml/kg) and overstretch (OS, 12 ml/kg) is shown with no evidence of neutrophil granulocyte infiltration to the alveoli when compared to no stretch (NS). Representative images are shown to confirm no infiltrating mononuclear cells in the alveolar space. Scale bar = 100 μm

To study the relationship between alveolar stretch and ER stress signaling we used ex vivo perfused and ventilated pig lungs. Heart and lung complexes were removed from healthy pigs and were perfused with 3% albumin containing tissue culture media to eliminate circulating immune cells. Subsequently animals were randomized to be exposed to no ventilation, Vt = 6 ml/kg (low stretch) and Vt = 12 ml/kg (overstretch) mechanical ventilation for 4 h with 5 cmH_2_O positive end expiratory pressure (PEEP) and 50% FiO2. These stretch settings were chosen to model lung protective (low-stretch) and injurious (overstretch) mechanical ventilation. This model allows the observation of the same physiological changes that take place in the alveoli during in vivo mechanical ventilation, but it has the key advantage of selectively studying parenchymal lung cell contribution to injury signaling. To comprehensively measure ER stress activation we performed western blotting for BiP, XBP-1, ATF6 and PERK in whole lung protein extracts (Fig. 1a). XBP-1 was chosen to study the downstream effects of the IRE-1α signaling activation. We observed increased PERK phosphorylation in response to overstretch but not to low-stretch. There was no significant change in BiP and XBP-1 expression in either stretch conditions. Both the total (p90) and the cleaved (p50) form of ATF6α, when compared to controls, showed significant decrease with overstretch (Fig. 1a). We also detected the total form of the ATF6β (p110) but not its cleaved from (p60). We found significant ISR activation by overstretch which was marked by the increase in eIF2α, ATF4, CHOP and GADD34. However low stretch did not alter ISR signaling. Quantified data is shown in Fig. 1b-i. To confirm the lack of inflammatory cells in the alveolar space hematoxylin-eosin stained lung histology slides are shown in Fig. 2. In the absence of infiltrating cells in the model, we concluded from these experiments that the lung parenchyma can exert ER stress signals independent of infiltrating inflammatory cells. Our data also show that the activation of ER stress signals is dependent on the magnitude of lung stretch.

### Mechanical overstretch activates PERK signaling in the alveolar epithelium

**Fig. 3:**
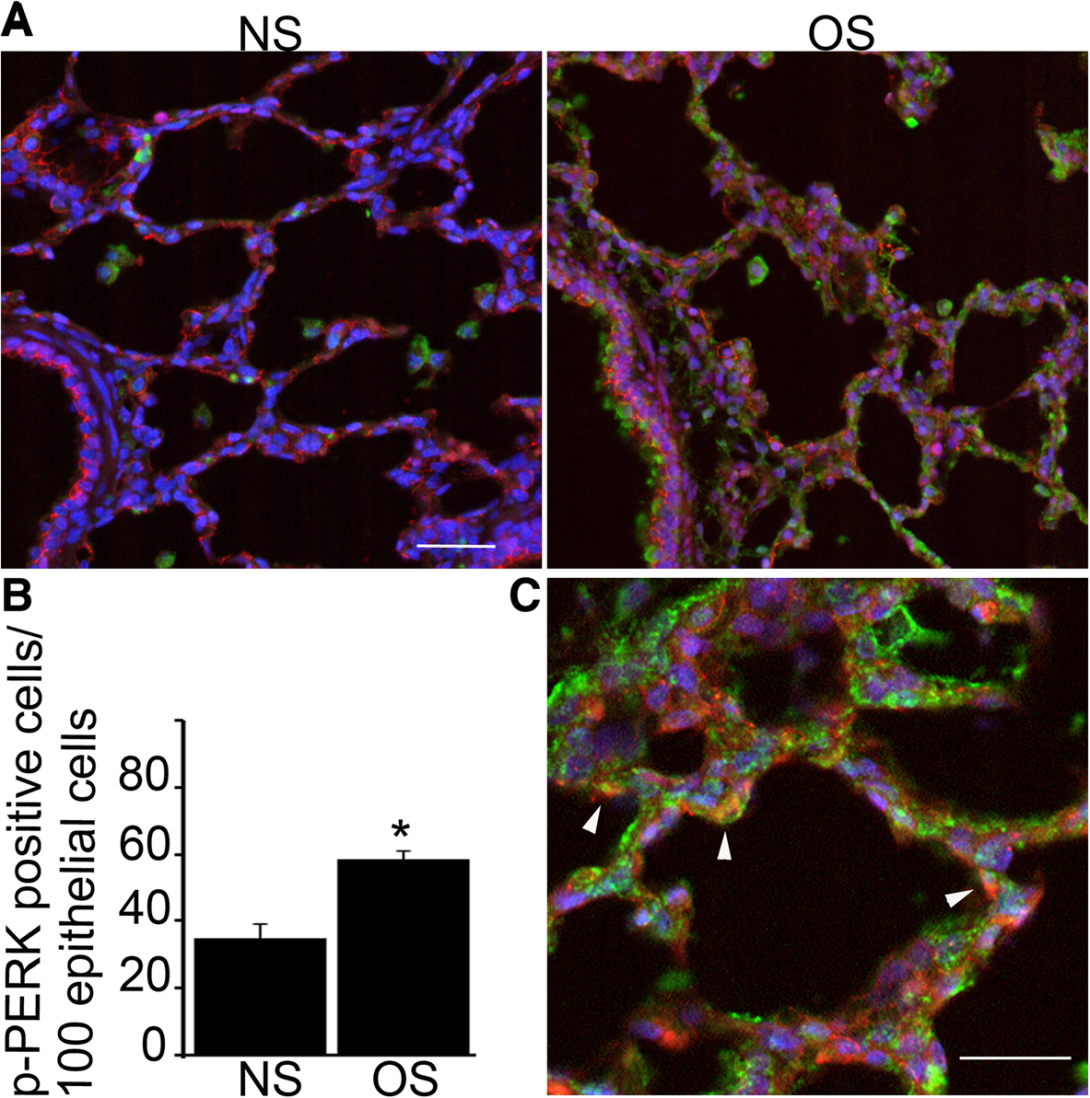
Mechanical overstretch activates PERK signaling in the alveolar epithelium. **a**. Perfused ex vivo lung complexes ventilated according to NS and OS protocol from Fig. 1 were used. At the end of the experiment lung tissue was incubated with green fluorescent-labeled p-PERK antibody and epithelial cells were labeled with red fluorescent conjugated epithelial- specific occludin antibody. OS significantly increased the number p-PERK positive cells, including those with dual p-PERK and occludin positivity. **b** The bar graph shows the number of p-PERK positive cells per 100 epithelial cells in NS and OS conditions. **c** High magnification image of the lung tissue shows p-PERK staining in occludin positive cells (arrows) confirming p-PERK localization in the epithelium. Green = green fluorescent-labeled p-PERK, Red = red fluorescent-labeled occludin, Blue = DPI for nuclear staining. Statistics: Two-way ANOVA was performed with Tukey-Kramer post hoc analysis. *N* = 4 animals/condition. Using laser scanning confocal microscope *N* = 5 random images/sample were taken for analysis. We count the number of p-PERK positive cells in 100 occludin positive cells per field and compared them between NS and OS conditions. Images were analyzed by FIJI program. *represents significant increase in the number of dual positive cells, *p* < 0.05. Data is presented as averaged values ±SEM. Image scale bar = 100 μm (A) and 20 μm (C)

We next evaluated PERK activation in alveolar epithelial cells in response to overstretch. We exposed pig lungs to overstretch or treated them as unstretched controls. Lung tissue was incubated with green fluorescent-labeled p-PERK antibody and we used red fluorescent conjugated occludin specific antibody to identify alveolar epithelial cells (Fig. 3a). Overstretch significantly increased the number of p-PERK positive cells and we quantified the number of p-PERK positive epithelial cells (Fig. 3b). Epithelial p-PERK localization is shown with high resolution imaging in Fig. 3c. These data suggest that overstretch activates PERK in the alveolar epithelium.

### PERK inhibition reduces VILI in vivo

**Fig. 4:**
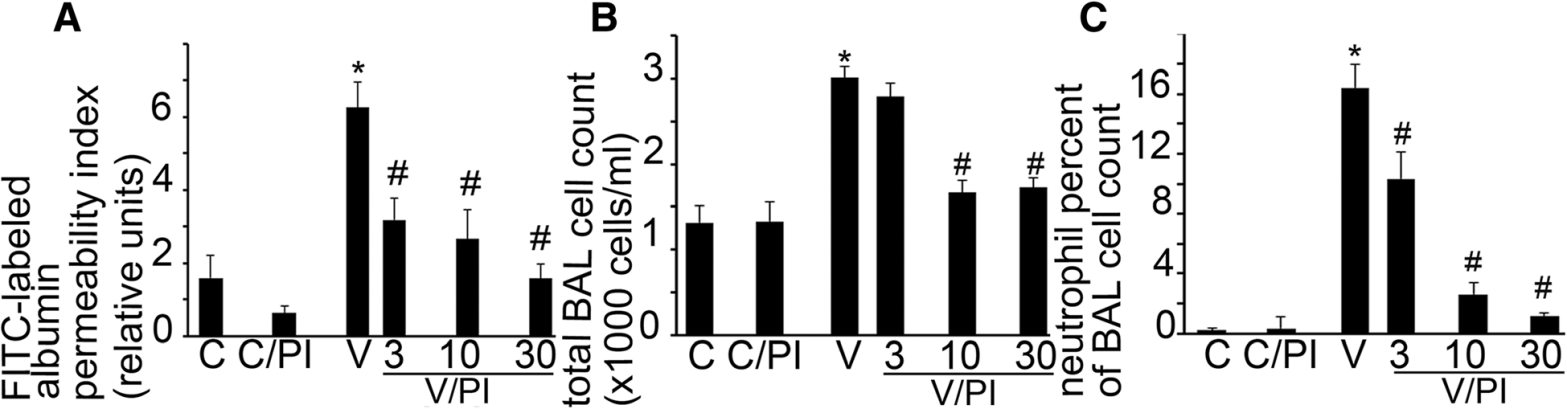
PERK inhibition reduces VILI in vivo**.** PERK. Rats were pretreated with increasing dosease of 3, 10 or 30 mg/kg GSK2606414 compound (PI) or its vehicle (0.1% TWEEN 80 in 0.5% hydroxyethyl-methylcellulose) via oral gavage. Four hours later the animals were anesthetized and mechanically ventilated (V) with 20 ml/kg tidal volume without postive end expiratory pressure or allowed to breathe spontaneously (**c**). Lung injury indices were compared among all groups. **a** PI treatment (V/PI) improved mechanical stretch-induced alveolo-capillary barrier dysfunction in a dose-dependent fashion. Lung injury parameters were not affected by PI in controls (C/PI). V/PI animals showed decreased fluorescent isothiocyanate (FITC)-labeled albumin permeability index when compared to V. **b**-**c** Mechanical stretch significantly increased BAL total and neutrophil cell count. Animals treated per V/PI protocol exhibited reduced infiltrating cell numbers when compared to V. Statistics: Kruskal-Wallis test was performed for multiple group comparison, and intergroup differences were analyzed with Wilcoxon rank sum test. *N* = 5–9 animals/condition. Data is presented as averaged values ±SEM.*represents significant increase in V vs. C conditions and #represents significant decrease in VPI vs. V conditions, *p* < 0.05

To confirm that PERK activation results in VILI, groups of rats were pretreated with 3, 10 and 30 mg/kg PERK inhibitor (PI) or its vehicle via gavage. Animals were subsequently ventilated with large tidal volume (Vt = 20 ml/kg) for 4 h to maximize injury or let to breathe spontaneously. Pulmonary edema formation was assessed with FITC-labeled albumin extravasation and inflammatory cell recruitment was measured in the BAL. PERK inhibition with moderate to high doses of PI (10-30 mg/kg) significantly reduced pulmonary edema formation (Fig. 4a), total BAL cell count (Fig. 4b) and neutrophil count (Fig. 4c). Low dose PI (3 mg/kg) was sufficient to reduce alveolo-capillary permeability and neutrophil recruitment to the alveoli (Fig. 4a, c). These results show that PERK inhibition improves VILI, and PI can be dosed to selectively study cellular contribution to alveolar injury.

### Mechanical stretch-induced ER Ca^2+^ efflux activates PERK

**Fig. 5:**
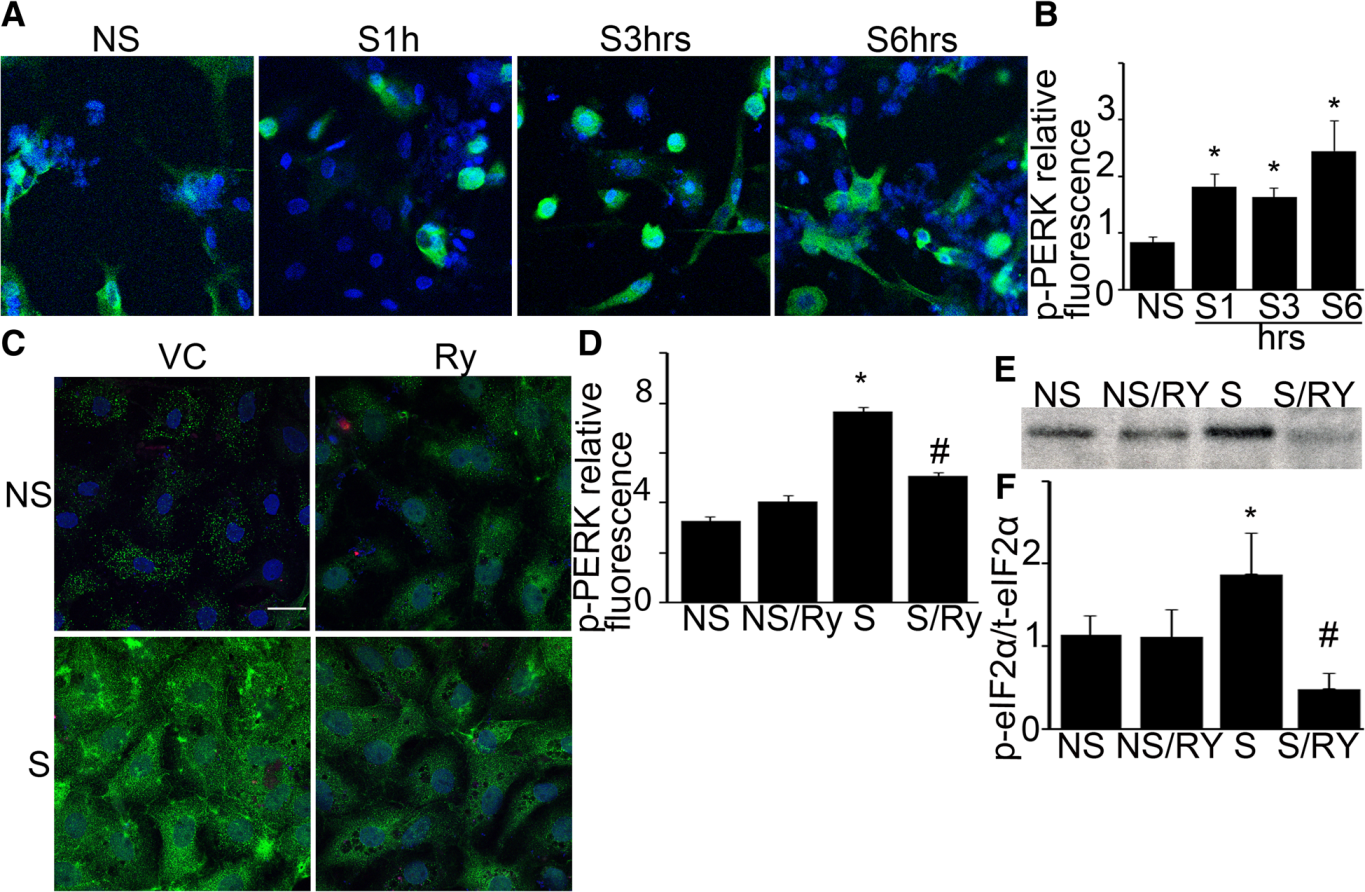
Mechanical stretch-induced ER Ca^2+^ efflux activates PERK in rat primary alveolar type-I like epithelial cells (RAEC-I). Freshly isolated rat epithelial cells were cultured for 4 days to form RAEC-I monolayers. **a** Monolayers were stretched for 1,3 and 6 h with 25% surface change (S) or used as unstretched controls (NS). At the end of the experiment fixed monolayers were treated with green fluorescent-labeled p-PERK antibody to detect PERK activation. Mechanical stretch significantly increased p-PERK at 1 h and it remained increased at 3 and 6 h. **b** Quantified data is shown. **c** Monolayers were pretreated with 20 μM Ryanodine (Ry) or its vehicle 0.01% DMSO (VC) for 1 h and subjected to biaxial stretch for 6 h with 25% surface change (S). Unstretched monolayers served as controls (NS). PERK activation was assessed with immunofluorescence staining as explained above. Mechanical stretch significantly increased p-PERK, which was significantly reduced by Ry pretreatment. Ry did not significantly change p-PERK in NS monolayers. **d** Quantified data is shown. **e** Western immunoblotting was performed to measure the effect of ER Ca^2+^ efflux blockade on eIF2α activation. Ryanodine treatment alone did not affect eIF2α (NS/Ry) compared to control (NS). Mechanical stretch (25%, 6 h) significantly increased p-eIF2α (S). Ryanodine treatment prevented stretch-induced eIF2α phosphorylation (S/Ry). **f** Quantified data is shown. Statistics: Two-way ANOVA was performed with Tukey-Kramer post hoc analysis for immunofluorescence studies. *N* = 4 biological monolayer replicates/condition, *p* < 0.05. Green fluorescent-labeled p-PERK signal was quantified using Fiji by threshold selection and average signal was calculated over 3 different fields of view. Data is presented as average signal ±SEM. Scale bar = 20 μm. For western immunoblot studies, Kruskal-Wallis test was performed for multiple group comparison, and intergroup differences were analyzed with Wilcoxon rank sum test. *N* = 4 biological monolayer replicates/condition, p < 0.05. Data is presented as averaged values ±SEM. *represents significant increase in green fluorescence S vs. NS conditions. #represents significant decrease between S and S/Ry conditions

We next explored a mechanistic link between epithelial PERK activation and ER Ca^2+^ signaling. We exposed RAEC-I monolayers to 25% biaxial stretch for 1,3 and 6 h. Unstretched monolayers served as controls. At the end of the experiment monolayers were incubated with green fluorescent labeled p-PERK antibody. The increase in p-PERK was detected with IF at each time point with stretch (Fig. 5a, b). The effect of ER Ca^2+^ release on PERK phosphorylation was studied by the pretreatment of monolayers with Ryanodine (Fig. 5c, d) at the 6-h time point. We have previously shown that 6 h mechanical stretch significantly increase epithelial barrier dysfunction and activates ISR signaling [13]. Ryanodine, at the 20 μM dose, blocks ER Ca^2+^ release. In the absence of Ca^2+^ efflux, PERK activation was significantly reduced, though not completely inactive (Fig. 5c, d). To evaluate if Ca^2+^ signal inhibition affects ISR activation downstream of PERK, we measured eIF2α phosphorylation in the presence and absence of stretch (Fig. 5e, f). Ryanodine treatment without stretch did not change p-eIF2α when compared to vehicle-treated controls. Mechanical stretch significantly increased p-eIF2α, which was reduced by Ryanodine (Fig. 5e, f). These studies provide new evidence that ER Ca^2+^ signals contribute to PERK and ISR activation in the alveolar epithelium.

### ARDS activates PERK and ISR signaling

**Fig. 6:**
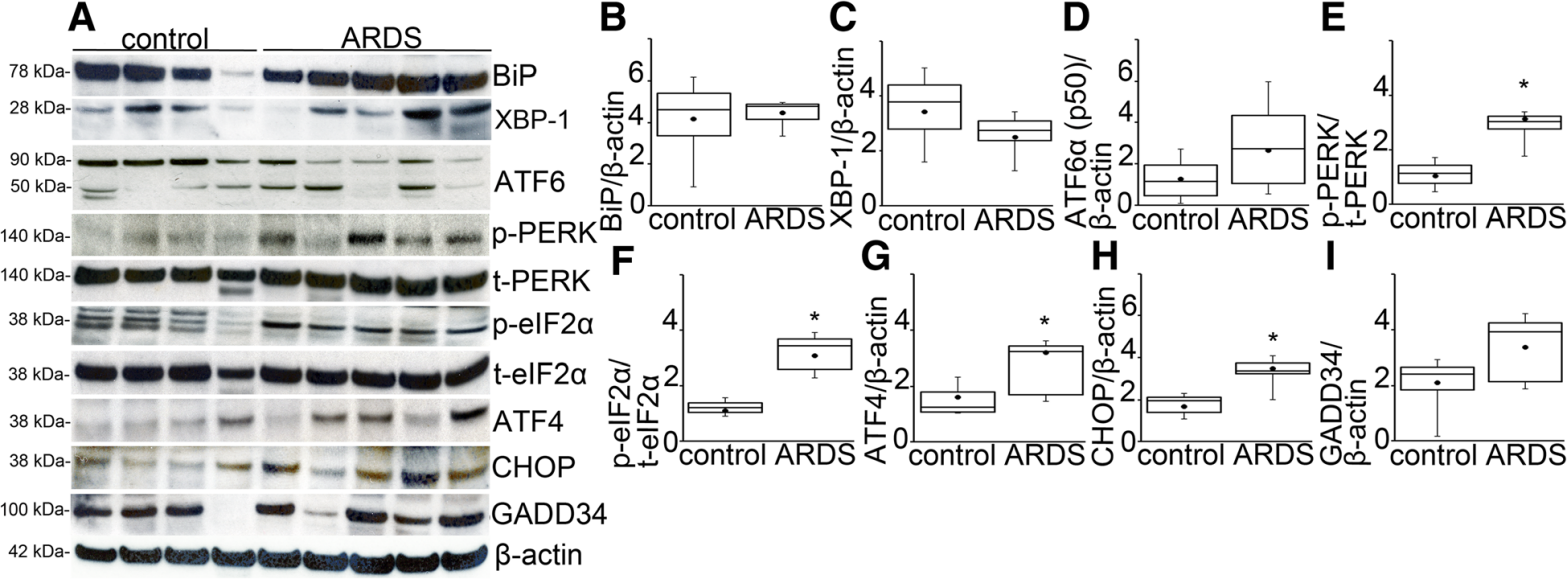
ARDS activates PERK and ISR signaling. **a** ER stress signaling was measured in protein extracts obtained from patients with ARDS and compared to non-ARDS (control). ARDS did not affect the expression of BiP,XBP-1and ATF6. We detected the phosphorylation of PERK, eIF2α and the increased expression of its downstream integrated stress response transcription factors ATF4 and CHOP. We did not detect significant change in GADD34 expression. We used total t-PERK for the loading control of p-PERK and t-eIF2 for p-eIF2α. All other protein expression was normalized to β-actin. **b**-**i** Quantified densitometry data is shown. Statistics: Kruskal-Wallis test was performed for multiple group comparison, and intergroup differences were analyzed with Wilcoxon rank sum test. Control samples *N* = 4, ARDS samples *N* = 5, *p* < 0.05. Data is presented as averaged values ±SEM in a five number summary format. *represents significant increase in p-PERK, p-eIF2α, ATF4 and CHOP in ARDS vs. control

To evaluate if increased ER stress signaling can also be observed in human ARDS, we used lung tissue from donors whose lungs were declined for transplantation (Fig. 6a). ARDS diagnosis was made post mortem by the presence of acute bilateral lung infiltrate on imaging within 1 week prior to death, a ratio of arterial oxygen partial pressure in mmHg to FiO_2_ less than 300 and a pathological evidence neutrophilic infiltrates or hyaline membranes in the alveolar space with or without septal edema. The clinical, radiological and histological characteristics of the patients are listed in Table 1. When compared to non-ARDS controls (samples 1–4), ARDS (samples 5–9) did not change the expression of BiP, XBP-1 and ATF6α (Fig. 6b). ATF6β was not detectable in the human lung tissue. We observed the activation of PERK, eIF2α and the increased expression of ATF4 and CHOP. We did not find significant expression change in GADD34 suggesting impaired deactivation of eIF2α in ARDS (Fig. 6a). Quantified data is shown in Fig. 5b-i. Based on these results, we concluded that PERK activation is a marker of lung injury in ARDS and PERK-mediated ISR signaling may contribute to the pathology of the disease.

## Discussion

Our study shows PERK ER stress signal activation in response to overstretch in the lung tissue. We have previously described that isolated alveolar epithelial cells respond to overstretch with increased PERK activation and downstream ISR signaling in rodent models [13]. Now we provide new evidences in a clinically relevant porcine VILI model and in human ARDS to show that PERK and ISR signaling has a critical role the pathology of acute lung injury. Alveolar cells are known to respond to injury stimuli by activating ER stress sensory pathways, which include PERK, IRE-1α, ATF6 and their upstream regulator BiP [23–25]. To evaluate if ER stress signaling is specific to PERK in response to mechanical overstretch, we measured BiP, IRE-1α transcription factor XBP-1 and ATF6 expression in our model. BiP expression did not change with stretch. This is consistent with findings in the lung injury literature [25] as BiP release of ER stress sensor molecules does not result in changes in its protein level [26]. We previously showed that high-strain mechanical stretch in RAEC-I cells activates PERK-induced ISR independent of IRE-1α [13]. XBP-1 level was also unaffected by overstretch, suggesting that injurious mechanical ventilation does not provoke a generalized ER stress signal in the alveolar space [14]. Interestingly, ATF6α expression significantly decreased with overstretch in ex vivo ventilated pig lungs. Little is known about role of ATF6 signaling in lung diseases. Increased ATF6 expression has been shown in animal models of asthma [27] and bleomycin-induced pulmonary fibrosis [24], but its role in VILI and ARDS has not been studied. ATF6 genes code for two protein isoforms (α or p90 and β or p110 forms), which are cleaved upon ER stress and translocate to the nucleus to activate pro-cell survival gene expression [28, 29]. We only detected the cleaved isoform of ATF6α but not β (Figs. 1 and 6) in our analysis, which is consistent with previous observations in the lung [24]. While the exact reason for ATF6α downregulation is not known, we can only speculate that this may represent the exhaustion of the ATF6 response [30]. ER stress signaling exhaustion has been previously described in response to continued ER stress [31].

Our findings suggest that there is a threshold to PERK activation by stretch. Overstretch but not low-magnitude (i.e. physiological) stretch resulted in the significant increase in PERK and ISR signaling, which suggest that injurious stretch is necessary to activate ISR-mediated cell injury. This is in concert with our previous observation in epithelial monolayer stretch, which showed that low magnitude stretch (12%) does not result in significant gene expression changes [32]. ISR and, in particular, CHOP play an important role in balancing cell fate. PERK activated phosphorylation of eIF2α is the critical step of ISR activation. Short activation of eIF2a leads to increased transcription of ATF4, but not CHOP, to promote cell recovery. Sustained eIF2α activation causes programmed cell death via CHOP-mediated signaling [33, 34]. Interestingly, CHOP-deleted mice are protected from hyperoxia-induced lung injury [25]. We do not have direct evidence of CHOP-mediated lung injury in our model, but our results show that inhibition of PERK, the major regulator of ISR and CHOP, reduces alveolar inflammation and permeability. Similar to the findings of Lozon et al. [25], our data (Fig. 1) indicate that there is baseline CHOP expression in the lung. Based on these findings, we speculate that physiological stretch results in low levels of CHOP expression to maintain alveolar homeostasis, but is insufficient to activate cell death.

Besides PERK, other kinases [25, 35–37] including General Control Nonderepressible 2 (GCN2), Protein Kinase R (PKR) and Heme-regulated Inhibitor (HRI) can also activate ISR via the phosphorylation of eIF2α. They respond to the specific stimuli of amino acid starvation (GNC2), double stranded RNA (PKR), heme deficiency (HRI) and oxidative stress (PKR and HRI). Compared to our model system, these stimuli require a longer time course (8 h to 2 months) to increase eIF2α phosphorylation, which makes it unlikely that they significantly contributed to stretch-induced ISR activation.

To connect PERK activation with lung injury signaling in vivo, in the absence of reliable genetic models [38], we used PERK inhibitor GSK2606414 (PI) [39, 40]. We have previously reported significant PERK inhibition with 30 mg/kg PI in lung tissue without cellular toxicity [13]. In the current study, we found that low dose (3 mg/kg) PI treatment prevented VILI-induced alveolar barrier dysfunction and neutrophil infiltration to the alveolar space. Higher dose (10 mg/kg) PI was necessary to effectively inhibit VILI-induced alveolar cell accumulation. This suggests that alveolar mononuclear cells are less sensitive to PI then parenchymal lung cells and neutrophils. Macrophages are essential mediators of VILI-induced inflammation [5] and have been reposted to express PERK [41]. Our findings imply that PI can be titrated to study cell specific contribution to PERK-mediated lung injury signaling.

The role of PERK in mediating mechanosensory signals was studied by subjecting RAEC-I cell to high-strain (25%) cyclic biaxial mechanical stretch. This stretch magnitude corresponds to approximately Vt = 15 ml/kg positive pressure ventilation [42]. Mechanical stretch increased p-PERK as early as 1 h and p-PERK remained activated at 6 h. Our laboratory and others have shown that Ca^2+^ influx from intracellular reservoirs and from the extracellular space can activate messenger molecules that regulate barrier function upon stretch in alveolar epithelium [43–45]. The ER represents a large source of Ca^2+^ and its release from the ER is a strong activator of PERK [46]. We have chemically inhibited Ca^2+^ efflux from the ER with 20 μM Ryanodine. At low concentrations (< 1 μM) Ryanodine selectively activates ER Ca^2+^ channels, while at high doses (20 μM) it inhibits them [47, 48]. Ryanodine was also chosen to block ER Ca2+ efflux because Ca chelators result in large shifts in ER Ca^2+^ levels and cause the activation of PERK [46]. The inhibition of Ca^2+^ efflux by high-dose Ryanodine significantly reduced stretch-induced PERK activation. These data provide evidence of a previously unknown Ca^2+^-dependent sensory function of PERK in the alveolar epithelium. While reduction of PERK activation was seen with inhibition of Ca^2+^ efflux, it did not completely block it. One possible explanation to persistent PERK activation is Ca^2+^ leak via damaged ER and cell membranes, which was suggested by Lumley et al. in response to significant cell stress [49]. The mode of interaction between PERK and Ca^2+^ requires further investigation. Jwa and colleagues showed that poly-ribosylation by Poly (ADP-ribose) Polymerase Family Member (PARP)-16 is critical for PERK activation. PARP16 has a C-tail in the ER lumen which is sensitive to ER Ca^2+^ efflux [50]. PERK ribosylation by PARP16 facilitates its dissociation from BiP [51], which eludes to a mechanism by which stretch-induced Ca^2+^ efflux activates PERK signaling. We further studied if Ryanodine inhibition of Ca^2+^ efflux impacts downstream ISR signaling and found that it also inhibits eIF2α phosphorylation. We have previously shown that PERK inhibition prevents stretch-induced eIF2α, ATF4 and CHOP activation [13]. This data suggest that stretch-induced Ca^2+^ signaling affects ISR via PERK.

To provide a human disease correlate to our pig ventilation data, we measured ER stress signaling in ARDS lung tissue and compared it to non-ARDS controls. Human samples were obtained from various common medical intensive care unit clinical scenarios which included pulmonary embolism, chronic obstructive pulmonary disease exacerbation, pneumonia and acute hypoxemic respiratory failure due to pulmonary fibrosis (Table 1).While these disease mechanisms may themselves activate ER stress signals our analysis showed that there is a superimposed ER stress signal with ARDS. A similar pattern of ER stress signal activation was seen in patients with ARDS to what was observed with mechanical overstretch in pig lungs except for ATF6α and GADD34. We did not observe differences in the expression of total and cleaved forms of ATF6α between control and ARDS human lungs. This data suggest that in human ARDS ATF6 is also regulated by mechanisms independent of stretch and circulating cells have a significant contribution to the overall expression of cleaved ATF6α. In a feedback loop, ATF4 and CHOP activate GADD34 which dephosphorylates eIF2α to prevent continuous ISR activation [16]. Persistent eIF2α phosphorylation has been previously reported in response to chronic disease [52], which implies that GADD34 response may be suppressed by prolonged ER stress. Taken together these data suggest that ER stress signaling is modulated by ARDS and that PERK activation may contribute to the mechanism of acute lung injury.

Our study raises two important questions. First, can ER stress signals help us to identify individual differences in ARDS development? Our human sample analysis suggests that the pattern of ISR activation may vary based on the etiology of the disease. This has important clinical implications as we are increasingly aware of the heterogeneity of ARDS and look for individualized treatment [53]. Second, how does ISR regulate alveolar homeostasis in VILI? Our preliminary data with PERK inhibitor suggest that PERK is an important regulator of alveolar barrier function and inflammatory signaling in the alveolar space. Crosstalk has been recognized between ISR activation and lung injury mechanisms [13, 25]. At this point, we can only speculate that ISR affects the protein structure of the epithelial barrier as it is sensitive to mechanical stretch [54].

Our study also has three major limitations. First, in the absence of healthy human tissue, we were unable to measure baseline ER stress signals in the lung. However we had access to ventilated lung tissue without significant acute injury and inflammation (Table 1). These samples served as proxy controls in which we were able to reliably exclude diffuse alveolar damage (DAD) and ARDS based on clinical criteria [55].Second, DAD is the signature pathological feature of ARDS but it is not seen in all cases [20]. To adequately diagnose ARDS, we completed retrospective analysis of clinical and radiology information available in PROPEL database and combined it with tissue histology evaluation. While the significant differences in ER stress signaling between ARDS and control samples are reassuring, it is still possible we misclassified samples. Third, our study is also limited by the absence of direct loss of function experiments. PERK gene deletion results in systemic inflammation and premature mortality in mice [38], which precludes us from performing VILI experiments on these animals. We are currently developing an epithelial specific PERK null mouse which will allow the direct testing of downstream modulators of lung injury.

**Fig. 7:**
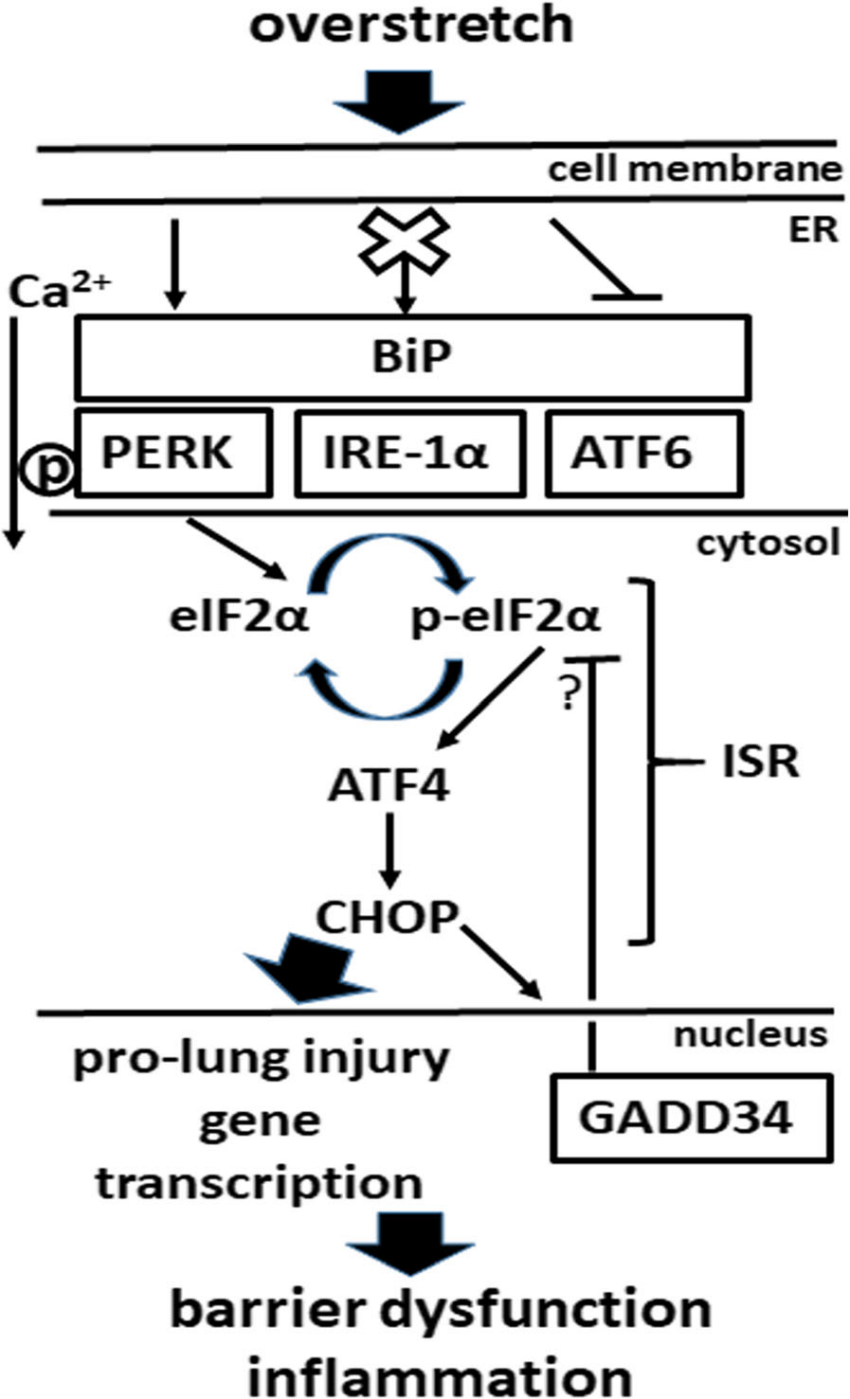
The hypothetical role of Protein Kinase R-like Endoplasmic Reticulum Kinase (PERK) signaling in VILI. Mechanical overstretch activates PERK endoplasmic reticulum (ER) stress signaling independent of Inositol Requiring Enzyme (IRE)-1α and Activating Transcription Factor (ATF)-6 in the alveoli. Stretch-mediated ER Ca^2+^ efflux contributes to PERK activation, which in turn increases Integrated Stress Response (ISR) by phosphorylating eukaryotic Initiation Factor (eIF)-2α and facilitating the transcription of ATF4 and CCAAT/Enhancer-binding Protein Homologous Protein (CHOP). Significant increases in CHOP expression result in pro-injury gene transcription leading to alveolar epithelial barrier dysfunction and inflammation. ISR-mediated Growth Arrest and DNA Damage-inducible Protein (GADD)-34 dephosphorylates eIF2α to prevent perpetual ISR activation. Arrow = activation, x = no effect, **|** = inhibition

The hypothetical role of PERK in VILI is shown in Fig. 7. Mechanical overstretch activates PERK ER stress signaling independent of IRE-1α and ATF6 in the alveoli. Stretch-mediated ER Ca^2+^ release contributes to PERK activation, which in turn increases eIF2α phosphorylation and the expression of ISR transcription factors ATF4 and CHOP. Significant increases in CHOP expression promotes pro-injury gene transcription leading to alveolar epithelial barrier dysfunction and inflammation. ISR-mediated GADD34 dephosphorylates eIF2α to prevent perpetual ISR activation.

## Conclusions

Our analysis of human, porcine and rodent lung tissue shows that PERK is a mediator of lung injury signals in VILI. Furthermore, we provide new evidence that PERK is a transmitter of epithelial stretch signals and PERK-mediated ISR signaling may contribute to the pathology of ARDS.

## Abbreviations

ARDS: Acute respiratory distress syndrome
ATF: Activating Transcription Factor-4 and 6
BAL: Bronchoalveolar lavage
BiP: Binding immunoglobulin Protein
Ca^2+^: Calcium ion
CHOP: CCAAT/Enhancer-binding Protein Homologous Protein
DAD: Diffuse alveolar damage
eIF: Eukaryotic Initiation Factor-2α
ER: Endoplasmic reticulum
FiO_2_: Fractional inspired oxygen
FITC: Fluorescent isothiocyanate
GADD: Growth Arrest and DNA Damage-inducible Protein-34
GCN2: General Control Nonderepressible 2
grs: Grams
HRI: Heme-regulated Inhibitor
IRE: Inositol Requiring Enzyme-1α
ISR: Integrated stress response
kgs: Kilograms
MEM: Minimal essential medium
p: Phospho
PEEP: Positive end expiratory pressure
PERK: Protein Kinase RNA-like Endoplasmic Reticulum Kinase
PI: PERK inhibitor
PKR: Protein Kinase R
PROPEL: Prospective Registry of Outcomes in Patients Electing Lung Transplantation
RAEC: I and II- rat alveolar epithelial type-I and II like cells
t: Total
VILI: Ventilator-induced lung injury
Vt: Ventilator tidal volume
XBP: X-box Binding Protein-1
μM: Micro molar
μm: Micrometer
